# THE DIMENSIONS OF YOUNG ONSET DEMENTIA (YOD) AND WHAT THESE MEAN FOR EXTENDED WORKING LIVES

**DOI:** 10.1093/geroni/igac059.494

**Published:** 2022-12-20

**Authors:** Philip Taylor

**Affiliations:** Federation University Australia, Victoria, Victoria, Australia

## Abstract

Drawing from a review of the literature and international statistical sources, this paper identifies the global prevalence of young onset dementia (YOD), its complex aetiology and its social and economic costs. YOD is often defined as dementia affecting those aged 65 or under, although definitions vary. Prevalence estimates also vary but recent evidence suggests a rate in the region of 119.0 per 100,000 population for those aged 30-64, and this is expected to increase markedly in the coming decades. Research identifies that the causes of YOD vary greatly among younger people. Individuals experiencing YOD face a complex range of psychological, familial, and socioeconomic challenges, while often being active in the labour market at the time of diagnosis. Costs of YOD cannot be easily disentangled from those for dementia generally but include: the direct costs of the ‘resources’ drawn on by the person with dementia; indirect costs, usually considered in terms of the value of lost productivity by the person with dementia or their carer, and less tangible costs, such as in terms of psychological wellbeing, social exclusion and time costs. The paper concludes by considering the potential significance of YOD for the future labour market in the context of ongoing efforts to prolong working lives.

